# miR-142-3p regulates autophagy by targeting ATG16L1 in thymic-derived regulatory T cell (tTreg)

**DOI:** 10.1038/s41419-018-0298-2

**Published:** 2018-02-19

**Authors:** Yunjie Lu, Ji Gao, Shaopeng Zhang, Jian Gu, Hao Lu, Yongxiang Xia, Qin Zhu, Xiaofeng Qian, Feng Zhang, Chuanyong Zhang, Hongbing shen, Keli L. Hippen, Bruce R. Blazar, Ling Lu, Xuehao Wang

**Affiliations:** 1 Liver Transplantation Center, First Affiliated Hospital, Nanjing Medical University, No. 300 Guangzhou Road, Jiangsu Province, Nanjing, 210029 China; 2 Department of Pediatrics, University of Minnesota Cancer Center, Minneapolis, MN 55455 USA

## Abstract

Thymic-derived regulatory T cell (tTreg) clinical trials show therapeutic promise in the prevention of acute graft-versus-host disease (GVHD) in allogeneic hematopoietic stem cell transplantation patients. However, strategies are needed to improve tTreg proliferative ability and survival as a means to improve tTreg therapy and reduce the requirement for producing large numbers of Treg cells for adoptive tTreg transfer. Autophagy is a self-degradative process for cytosolic components, which is involved in cells death, differentiation, lymphocyte homeostasis, and tTreg function. Studies have shown that mice with tTreg cells that have a disrupted autophagy process have defective tTreg cell generation and function, resulting in autoimmune disease and failed GVHD prevention by adoptively transferred tTreg cells. We found the attenuated autophagy status during ex vivo expansion, which leads us to determine whether tTreg cell survival could be augmented by miR-142-3p, the miRNA which is highly expressed in tTreg cells and potentially targets autophagy-related protein (ATG)-1, ATG16L1. We demonstrate that miR-142-3p downregulates ATG16L1 mRNA and production of ATG16L1, that has been linked to autoimmune diseases. Conversely, miR-142-3p knock-down improved tTreg cell expansion, survival and function in vitro and vivo. In aggregate, these studies provide a new approach that uses miR-142-3p knockdown to increase tTreg cell efficacy by increasing ATG16L1 mRNA and protein and the autophagy process.

## Introduction

CD4^+^CD25^+^CD127^low^FOXP3^+^ thymic-derived regulatory T cells (tTreg) are necessary for the maintenance of immune homeostasis. Clinical trials of Treg cells aim to reduce or replace the use of immunosuppressive drugs, which is needed lifelong medication and might cause significant side-effects. So far Treg treatment has been proved to be an efficient way to reduce the incidence and severity of graft-versus-host disease (GVHD) in transplantation patients^1^. Additional clinical trials have confirmed the potential therapeutic properties of Tregs, and long term self-tolerance could be induced by injected Tregs through a process of “infectious tolerance” without immunosuppressive drugs^1^. Although achieved several methods have been developed to improve tTreg function, there are few publications which focus on tTreg proliferative ability and survival, important in preventing GVHD or autoimmune disease^2,3^.

Autophagy is a self-degradative process for cytosolic components, which is connected to cell survival pathway with nutrient recycling during starvation. Multiple cellular death process including several aspects of immunity are caused by autophagy^4–6^. Moreover, autophagy can favorably impact antigen processing, lymphocyte homeostasis, and cytokine secretion in immune responses^7–9^. Thus, autophagy is indispensable for cell homeostasis and survival mechanism. The autophagy-related protein (ATG) family is suggested to control T cell activation, proliferation and survival^10^. Autophagy-related protein 16-1 (ATG16L1) contributes a critical role in autophagy and ATG16L1 dysfunction leads to immune diseases such as Crohn’s Disease and decreased antibacterial defense^11,12^. Since autophagy-dependent tTreg cells are critical for the control of GVHD^13^, we hypothesized that targeting ATG may improve tTreg survival.

MicroRNA (miRNA) are small non-coding RNA molecules that can either target mRNA transcription or mediate posttranscriptional gene repression^14,15^. miRNAs are implicated in cell proliferation, survival, and function though an integrated signaling network. One such miR, miR-142-3p, is known to negatively regulate T cell activation in systemic lupus erythematosus (SLE) patients and hence may be a candidate for miR targeting^16^. In our previous study using TaqMan Low Density Array, we found that miR-142-3p was the second most highly differentially expressed miRNA in ex vivo expanded human tTreg cells as compared to naïve T cells^17^. Thus, we sought to determine whether miR-142-3p controls tTreg biological properties such as proliferation, survival, and suppressor function. We show that miR-142-3p regulates these tTreg function by targeting autophagy through ATG16L1 mRNA downregulation, and conversely that miR-142-3p knockdown improves tTreg survival and function as assessed both in vitro and vivo.

## Materials and methods

### Mice

NOD/SCID/mice were purchased from The Beijing Vital River Laboratory, and housed in a specific pathogen-free facility in micro-isolator cages. Mice were used at 8–12 weeks. Animal protocols were approved by Nanjing Medical University.

### Cell purification and culture

Peripheral blood (PB) leukapheresis products were obtained from volunteers in Nanjing Medical University. Naïve human PB tTreg (CD4^+^CD25^+^CD127^−^) were sort-purified from PB mononuclear cells (PBMNCs) (Ficoll-Hypaque, Amersham Biosciences) in a two-step procedure.

tTreg cells were stimulated with anti-CD3/CD28 mAb-coated Dynabeads (Life Technologies, Carlsbad, CA) at 1:3 (cell to bead) ratios in the presence of recombinant IL-2 (300 U/ml) (Chiron, Emeryville, CA) in X-Vivo-15 (BioWhittaker, Walkersville, MD) media supplemented with 10% human AB serum (Valley Biomedical) on day 0. Cells were counted and cultured at the concentration of 0.5 × 10^6^ cells/ml and IL-2 (300 U/ml) was renewed every 2 or 3 days. On point days (day 0 or 14), cells were re-suspended at 0.5 × 10^6^ cells/ml and treated with antagomir or agomir and renewed together with IL-2. Cells were harvested and assayed as listed.

### Flow cytometry, imagestream, and antibodies

Human-specific antibodies used for flow cytometry included: CD4(RPA-T4), CD8((RPA-T8), CD25(M-A251), CD45RA(HI100), Annexin V(PE), 7-AAD(FITC) were purchased from BD Pharmingen, while FoxP3 (clone 249D) is from BioLegend and Ki67 is from eBioscience. The annexin V (PE)/7-AAD(FITC) were applied to assess the apoptosis of tTreg. Acquisition was performed using a CATON (BD Bioscience) and data were analyzed using FlowJo software (TreeStar).

### Immunofluorescence confocal microscopy

Confocal microscopic analysis was performed to examine the cellular autophagy level in cells stably transfected with mRFP-LC3-GFP lentivirus. Briefly, cells were infected by autophagy detection lentivirus expressing SensGFP-StubRFP-LC3 for 48 h and reached 60–80% confluence, then were washed with PBS buffer, cells are then cultured in 96-well-flat bottom plates at a concertation of 5 × 10^3^ cells per well in media in the presence of IL-2 as above, the appropriate concentration of the antagomir or agomir were added for 2 more days. Then we scan cells in CQ1 using SensGFP, StubRFP, and Hochest three-channel immediately, and analysis the data by “see colour dots in cellbody” mode in CQ1.

Confocal microscopy (CarlZeiss LSM710, Germany) was used to analyze cellular autophagy level by the laser scanning performed with excitation at 488 nm and 561 nm wavelengths, respectively. The ImageJ software was used to analyze all quantitative images. To quantify the autophagic vacuoles, the background of images in the same series was filtered by the same threshold. Then, the “analyze particle” option of ImageJ was applied to quantify the mean fluorescence intensity, indicating the autophagy activity^18^.

### Anti- and pro-apoptotic gene expression analysis and TLDA (Taqman Low Density Arrays) assay

RNA was extracted from cell pellets using RNeasy Mini Kit with on-column DNase digestion (Qiagen; Hilden, Germany). cDNA synthesis was performed as described in the Expression Analysis Technical Manual (Affymetrix; Santa Clara, CA). GAPDH was used as control gene and related gene expression (Bcl-2, Mcl-1, Bcl-xL, BID, and BAX), (all from IDT, Coralville, Iowa) was analyzed on an Applied Biosystems 7500 Real-Time PCR System using Taqman Universal PCR Master Mix #4304437 and Assay on Demand primer/probe kits (Applied Biosystems; Waltham, MA). For TLDA assay, TLDA v2.0 was performed on the 7900HT real-time PCR system (Applied Biosystems) according to the manufacturer’s protocol. Average delta CT was acquired from the results for further analysis. PCR cycling conditions were performed as follows: 95 °C for 15 s and 60 °C for 1 min, 40 cycles and then 95 °C for 10 min. To normalize RNA input, Human RNU44 small RNA was used as an internal control.

### miRNA target prediction and luciferase reporter assay

Potential miRNA targets were sorted by utilizing miRNA prediction software TargetScan (targetscan.org), MIRDB (http://www.mirdb.org/) and microRNA (http://www.microrna.org/). For the luciferase reporter assay, the pGL3 firefly luciferase reporter plasmids with the WT or MUT 3′ UTR sequences of ATG16L1 were transiently transfected into HEK293 cells along with 25 nM miR-142-3p precursor or negative control precursor and a Renilla luciferase reporter for normalization. After 2 days, the luciferase activities were measured by Dual-Luciferase® Reporter Assay System. Based on the cells transfected by pGL3 control vector, the mean of the results was set as 100%. Data are mean and standard deviation (SD) of separate transfections.

### Suppression assays

The in vitro–suppressive capacity of expanded tTregs was assessed with a CFSE (carboxyfluorescein succinimidyl ester) inhibition assay as previously published^19^. Briefly, PBMNCs were purified, labeled with CFSE (Invitrogen), and stimulated with anti-CD3 mAb-coated beads (Dynal) ± cultured tTreg (1:2–1:32 tTregs/PBMNCs). On day 4, cells were stained with antibodies to CD4 and CD8 and suppression was determined from the Division Index (FlowJo, TreeStar). nTregs suppressed CD4 and CD8 T cell responses equivalently and only CD8 data are presented.

### Xenogeneic GVHD model

NOD/Scid mice between 8–12 weeks old were housed in a pathogen-free facility in micro-isolator cages. On day 0, mice were irradiated with 50 cGy. Human PBMNCs (10 × 10^6^) were injected with or without expanded tTregs (10 × 10^6^). Mice were assessed for signs of GVHD daily, weighed thrice weekly, and human cells in blood quantitated by flow cytometry on the specified dates.

### Statistical analyses

RT-PCR data were analyzed using SDS v2.3 software. Survival data were analyzed using Prism 5 (Mantel-Cox). Other data were analyzed by analysis of variance (ANOVA) or Student *t* test. Probability (*P*) values less than or equal to 0.05 were considered statistically significant.

## Results

### tTreg autophagy status and ATG protein levels change in the process of expansion

**Fig. 1:**
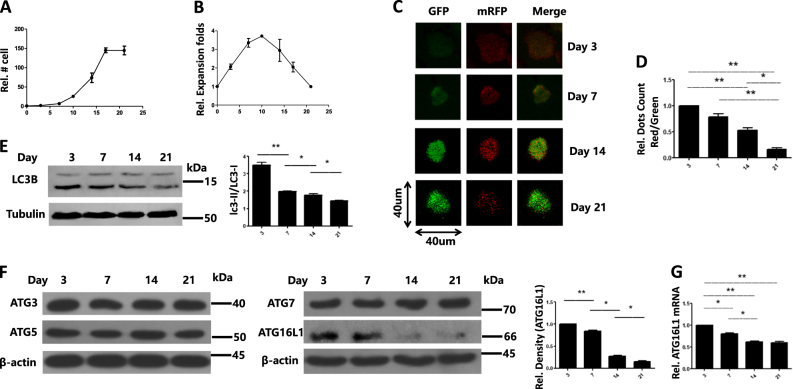
Attenuated tTreg autophagy status and apoptosis-related gene changes during ex *vivo* tTreg expansion. (*n* = 3) . CD4+CD25+CD127− tTreg cells were sort-purified by MACS and stimulated with anti-CD3/28 beads in the presence of IL-2 as indicated in methods part. **a** Cell number and **b** Relative expansion folds were recorded every 2–3 days until day 21. **c** Immunofluorescence confocal microscopy test for tTreg autophagy status: cells were transfected with mRFP-LC3-GFP lentivirus for 48 h, representative cellular autophagy level by the laser scanning from day 3 to 21, respectively. **d** The ratio of punctate autophagic vacuoles was measured by ImageJ software. **e** LC3B western blot results to show the autophagy status in tTreg culture process. **f** ATG3/5/12/16L1 expression by western blot in tTreg culture process. **g** RNA was purified and qRT-PCR used to determine the ATG16L1 mRNA expression Values indicate mean ± SEM of these experiments. (*P < 0.05 and **P < 0.01)

Initially, we sorted CD4 + CD25 + CD127-tTreg cells (purity ≥ 94%) from healthy donors and then stimulated them with anti-CD3/28 beads in the presence of IL-2 (renewed every 2 days) on day 0. We monitored cell number and expansion in 21 days after stimulation and found that in the culture process of tTreg, the fold expansion peaked around day 10 to 14 and then decreased with time (Figs. 1a,b). Although levels of the tTreg master transcription factor, FoxP3, remained stable in cultured cells (data not show), these tTreg cells did exhibit a lower autophagy status over time. We transfected mRFP-LC3-GFP lentivirus into tTreg cells at different time points (day 0, 4, 11, and 18) and tested the autophagy levels at different time points, respectively (day 3, 7, 14, and 21). After cell transfection and when cells reached 60–80% confluence, we analyzed the total number of autophagosomes and autolysosomes (mRFP+, GFP+) vs autophagosomes only (mRFP−, GFP+). This ratio was calculated, which reflects the levels of autophagy. As shown in Fig. 1c,d, the autophagy flow was decreased with time (red vs green), which showed similar tendency with expansion. LC3B western blot results in Fig. 1e also demonstrated the attenuated autophagy level from day 3 to 21.

ATGs, such as ATG3, ATG5, ATG7, and ATG16L1^20–24^, are necessary for maintaining the frequencies and numbers of peripheral CD4+ and CD8+ T cells. Therefore, we showed the ATG protein levels by western blot. The results in Fig. 1f suggested that only ATG16L1 changed in the process of culture. We next further quantified mRNA expression for ATG16L1 mRNA using qRT-PCR. Deficiency of ATG16L1 leads to decreased T cell numbers in the spleen and mLN in *ATG16L1*^ΔCD4^ mice^22^. Taken together, tTreg cells show attenuated autophagy status with decreased ATG16L1 expression in the process of expansion culture, which might account for the lower fold expansion over time.

### miR-142-3p negatively regulates ATG16L1 expression in tTreg cells

**Fig. 2:**
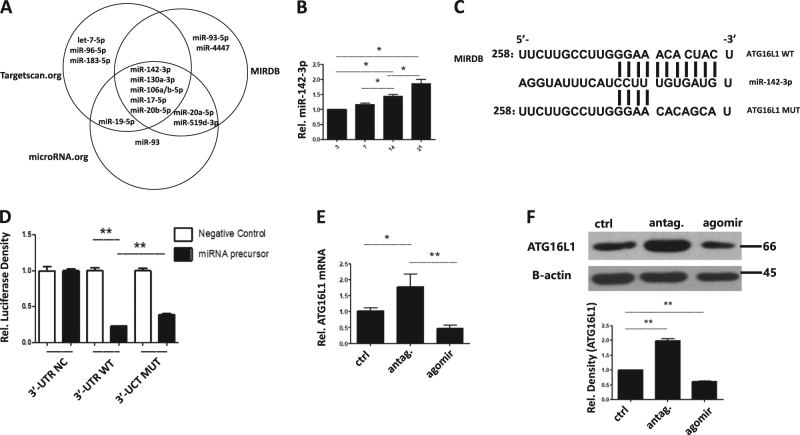
ATG16L1 is a direct target of miR-142-3p and knock-down of miR-142b-3p increased ATG16L1 expression in human tTreg cells. **(*n*** = **3).** To assess whether human miR-142b-3p targets ATG16L1 mRNA, HEK293 cells were transduced with plasmids carrying wild type (WT) or mutant (MUT) 3′UTR sequences from ATG16L1 linked to a luciferase reporter gene. Cells were also transfected with a Renilla luciferase reporter construct for normalization. **a** 3 software (targetscan.org, MIRDB and microRNA. org) were utilized to predict the potential mRNAs which targets ATG16L1 mRNA, miR-142-3p was involved in tTreg function with highest possibilities. **b** RNA was purified and qRT-PCR used to determine the expression of **b** miR-142-3p. **c** Schematic representation of the miR-142-3p target sequence within the 3′UTR of *ATG16L1*. Two nucleotides (complementary to nucleotides 6 and 8 of miR-142-3p) were mutated in the 3′ UTR of ATG16L1. The numbers indicate the positions of the nucleotides in the reference wild-type sequences. **d** Activity of the luciferase gene linked to the wild type (WT) or mutant (MUT) 3′UTR of *ATG16L1*. Luciferase activities were measured after 48 h. The mean of the results from the cells transfected by control vector was set as 100%. The data are mean and standard deviation (SD) of separate transfections (*n* = 3). Naïve PB tTreg were sort-purified, expanded in vitro, and were treated miR-142-3p antagomir or agomir as previously described. After treatment, cultured cells were assessed for ATG16L1 mRNA and protein expression by RT-PCR or western blot (**e** and **f**, respectively). Values indicate mean ± SEM of these experiments. (*P < 0.05 and **P < 0.01)

Based on previous studies, deletion of Atg16l1 in T cells results in spontaneous intestinal pathology with decreased Treg differentiation^22^. Both miR-142-3p/ATG16L1 genetic variations are associated with inflammatory bowel diseases (IBD)^25^, next we tried to find if ATG16L1 expression is associated with miR-142-3p level in tTreg cells. Based on our previous studies in which we compared miRNA expression profiles between ex vivo expanded tTreg and conventional T cells, we identified the top ten differentially expressed miRNAs, of which miR-142-3p was the second mostly highly upregulated in tTregs vs T cells^17^. We sought to determine whether miR-142-3p controls tTreg biological properties such as proliferation, survival, and suppressor function. We utilized miRNA target prediction software TargetScan (targetscan.org), MIRDB (http://www.mirdb.org/) and MiRNA (http://www.microrna.org/) to identify putative miRNAs for ATG16L1 mRNA downregulation. Five miRNA candidates were found (Fig. 2a). Of these only miR-142-3p was noted to be differentially expressed in our prior screen^17^. We next tested these five miRNAs in ex vivo expansion cultures. The expression of miR-130a-3p and miR-106a/b-5p were too low to be detected in tTreg cells, while miR-17-5p and miR-20b-5p showed no change in the process of expansion (data not shown). As suggested in Fig. 2b, expanding tTregs increased miR-142-3p expression, which might be related to attenuated ATG16L1 expression. The 6–8 nucleotides (seed region) at the 5′-end of the miRNA binds to 3′-UTR of target mRNA. Therefore, we structured wild-type (WT) or mutated (MUT) 3′-UTR sequences of ATG16L1 based on potential binding sites predicted by the software mentioned above (Fig. 2c) and used pGL3 firefly luciferase reporter plasmids to transiently transfect HEK293 cells. To test this hypothesis, an ATG16L1 luciferase reporter assay was employed to reveal potential binding sites of miR-142-3p. These results support our hypothesis that miR-142-3p can directly target ATG16L1 mRNA. As shown in Fig. 2d, 3′-UTR-NC control with unloaded plasmid, 3-UTR-WT and 3′-UTR-MUT groups were treated with miR-142-3p mimic. As expected, WT groups demonstrated significant decreased luciferase activity, and MUT group showed up-regulation compared to WT (Fig. 2d). We further tested this effect in tTreg cells. Agomir or antagomir was added in tTreg cells on day 14 and after 2 days and ATG16L1 mRNA and protein were tested. As shown in Fig. 2e, overexpression of miR-142-3p decreased ATGL1 mRNA while the inverse effects were detected after antagomir treatment. In accord with mRNA levels, miR-142-3p inversely correlated with ATG16L1 protein expression in tTreg cells (Fig. 2f). Thus, we conclude that miR-142-3p negatively regulates ATG16L1 expression in ex vivo expanded human tTreg cells.

### miR-142-3p—ATG16L1 axis controls tTreg function by regulating FoxP3 expression and proliferation in vitro

**Fig. 3:**
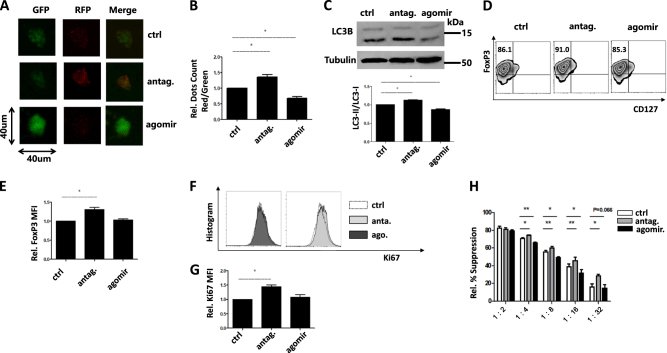
tTreg cells treated with miR-142b-3p antagomir show enhanced autophagy status, FoxP3 expression, with suppressive function. **(*n*** = **3).** PB CD4+CD25+CD127− tTregs were sort purified by MACS, expanded in vitro for 14 days, treated with or without agomir/antagomir for 2 more days. Immunofluorescence confocal microscopy test for tTreg autophagy status after treatment. **a** Representative cellular autophagy level by the laser scanning. **b** The ratio of punctate autophagic vacuoles was measured by ImageJ software. **c** LC3B western blot results to detect the autophagy status after antagomir or agomir treatment. **d**) Representative example of Foxp3 vs CD127 staining on tTreg treated with antagomir or agomir (gated on CD4+ cells). Summary of overall **e** level of Foxp3 expression and **f** representative example of Ki67 histogram on tTreg treated with antagomir or agomir (gated on CD4+ cells). Summary of overall **e** level of Ki67 expression after antagomir/agomir treatment. **g** Percent suppression of in vitro, anti-CD3–mediated CD8+ T cell proliferation at ratios from 1:2 to 1:32 (tTreg: PBMC) as determined by CFSE dye dilution. Values indicate mean ± SEM of these experiments (*P < 0.05 and **P < 0.01)

**Fig. 4:**
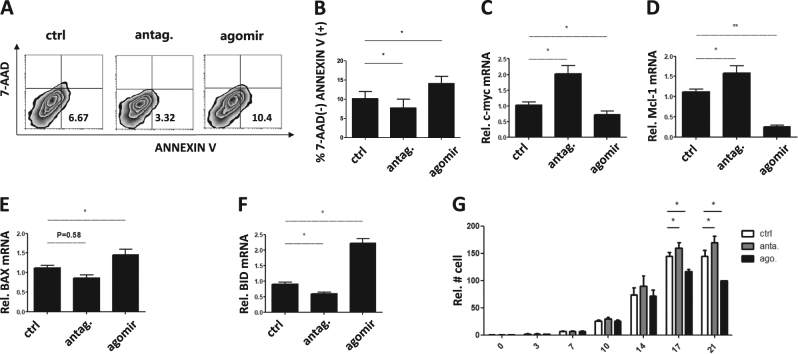
Treatment with miR-142-3p antagomir increases cell viability, proliferative ability, anti-apoptotic and decreases pro-apoptotic gene expression and enhances tTreg persistence and expansion. (*n* = 3). tTreg were sort purified, expanded in vitro and were either left untreated, or were incubated with agomir or antagomir. **a** Representative example of 7AAD vs ANNEXIN V staining on tTreg treated with antagomir or agomir (gated on CD4+ cells). Summary of overall **b** % ANNEXIN V+ 7-AAD− cells in tTreg from each group. Following treatment, RNA was purified and qRT-PCR used to determine the expression of anti-apoptosis genes **c** c-Myc, **d** Mcl-1, and pro-apoptotic genes **e** BAX, **f** BID. **g** Cell number was recorded every 3–4 days until day 21 and since day 17 antagomir group shows better while agomir group shows worse cell survival compared to control (*P* < 0.01 and *P* < 0.1, respectively). Values indicate mean ± SEM of these experiments. (*P < 0.05 and **P < 0.01)

Since expansion ability of tTreg cells decreased after day 14, we added antagomir or agomir on day 14 and harvested them for further assays on day 16 to see if their suppressive and proliferative phenotype could be affected by antagomir/agomir. Fluorescence confocal microscopy was used to confirm whether the ATG16L1 autophagy effects are controlled by miR-142-3p. As shown in Fig. 3a,b, knock-down of miR-142-3p with an antagomir strengthened autophagy activity, while agomir treatment reversed this effect. LC3B western blot results in Fig. 3c also comfired strengthen autophagy levels after antagomir treatment. Previous study shows that ATG16L1 plays an essential role in the maintenance of FoxP3 in Treg cells^22^. We quantified FOXP3 protein expression and in vitro suppressor function after treatment. As might be anticipated for the high differential expression of miR-142-3p in ex vivo expanded tTregs vs conventional T cells, we found that FOXP3 mean fluorescence intensity was negatively regulated by miR-142-3p and increased by antagomir exposure (Fig. 3d,e). Interestingly, we did not see that treatment with a miR-142-3p agomir decreased FoxP3 expression, which may indicate that endogenous miR-142-3p are sufficient to achieve maximal downregulation. We next monitored Ki67 expression, indicative of proliferation. Knock-down of miR-142-3p with an antagomir did increase Ki67 expression (Figs. 3f and 4g). Similar to FoxP3 levels, overexpression of miR-142-3p did not change Ki67 expression compared to control group. We also confirmed the stronger suppressive function after antagomir/agomir treatment in vitro (Fig. 3h), which is in accordance with FoxP3 expression. Therefore, the suppressive function of tTreg could be enhanced by miR-142-3p antagomir and miR-142-3p—ATG16L1—FoxP3 pathway plays a necessary role in vitro.

### Knock-down of miR-142-3p improves tTreg survival and promotes cell expansion

Although FoxP3 showed no differences between control and agomir groups, we did see the decreased suppressive function after agomir treatment, which suggests that tTreg cell survival might be also important for suppressive function. To determine whether increased miR-142-3p levels are related to attenuated viability, proliferative ability or survival during ex vivo tTreg expansion, tTregs on day 14 were treated with a miR-142-3p agomir or antagonist for 2 days. Early apoptosis was measured by Annexin-V staining. Overexpression of miR-142-3p induced early apoptosis in tTreg cells while antagomir treatment delayed apoptosis (Figs. 4a,b). tTreg-related anti/pro apoptosis genes (Bcl-2, Bcl-xL, Mcl-1, c-myc, BAX, BID, and BAD) play a key role in cell survival^26–29^. Of them, Mcl-1, c-myc, BAX, and BID expression were related to cell autophagy, which is essential for cell cycle and function^30–32^. Therefore, we qualified these genes expression by RT-PCR. Higher expression of anti-apoptosis gene (Mcl-1 and c-myc) and lower expression of pro-apoptosis gene (BAX and BID) was seen with antagomir compared to control group (Fig. 4c-f) while agomir treatment showed opposite results. To conclude, miR-142-3p negatively regulates tTreg apoptosis status. Finally, to confirm the effects of miR-142-3p on proliferation, we sorted fresh CD4+CD25+CD127− tTreg cells by MACS and treated them with agomir or antagomir on day 0 (renewed with new media). We found an increased cell number on days 17–21 of culture in antagomir group as compared to control, whereas cell number was decreased in agomir group (Fig. 4g,h), which might due to decreased apoptosis and increased anti-apoptosis genes expression. We conclude tTreg cells in agomir group were driven into apoptosis process while antagomir treatment prolonged cell survival with stronger proliferative ability. These results indicate that miR-142-3p negatively regulates tTreg ex vivo expansion, viability, and survival.

### Knock-down of miR-142-3p antagomir treatment significantly prolongs the survival and Treg persistence in a xenogeneic model of GVHD

**Fig. 5:**
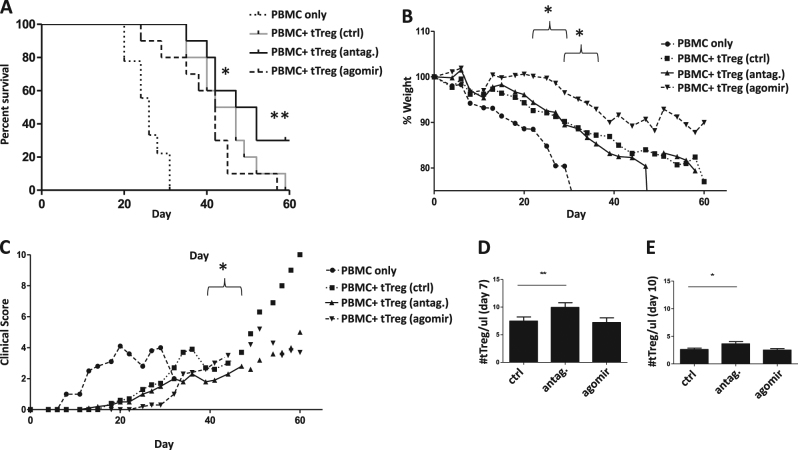
Antagomir treated tTregs decrease mortality in a xenogeneic model of GVHD. Naïve PB tTreg were sort purified, expanded in vitro and were either left untreated, or were incubated with scramble RNA or miR-142-3p antagomir for 2 days. Following treatment, tTreg were washed and co-transferred (10 × 10^6^) with allogeneic PBMC (10 × 10^6^) into NOD/Scid mice to assess the ability to ameliorate xenogeneic GVHD. For groups PBMNCs, control, antagomir, and agomir, *n* = 10, 10, 10 and 10, respectively. **a** Kaplan-Meier survival curves for mice receiving PBMC ± groups of tTreg (*P < 0.05). **b** Average weight (percentage of initial) for mice surviving on a given day for different groups of mice (*P < 0.05 for all tTreg groups from days 0 to 61). **c** Average GVHD score for mice surviving on a given day for different groups of mice (*P < 0.05 for all tTreg groups from days 0 to 60. Mice were sacrificed for blood, then tTreg number (/ul blood) was measured and recorded by HLA-mismatch flow assay on **d** day7 and **e** day 10 (*n* = 3, respectively). Data shown are representative of two independent xGVHD experiments

Knock-down of miR-142-3p in ex vivo expanded tTregs upregulated ATG16L1 expression and such tTreg cells showed stronger FoxP3 expression and suppressive function in vitro. To determine whether these findings could be harnessed to improve the GVHD protective effect of tTreg adoptive transfer, ex vivo expanded for 14 days tTregs (10 × 10^6^) were left untreated or treated with agomir or antagomir for 2 days, then washed and injected with PBMCs (10 × 10^6^) into immune deficient NOD/SCID mice. As shown in Fig. 5a, all three groups of mice receiving tTreg had significantly reduced GVHD-induced lethality compared to PBMC-only controls (*P* < 0.01, 0.001, and 0.01 for control, antagomir and agomir treated tTreg, respectively). Consistent with vitro results, mice receiving antagomir-treated tTreg had significantly increased survival compared to mice receiving untreated or agomir treated tTregs (*P* < 0.01 and 0.01 for control and agomir tTreg, respectively) while agomir group showed even poorer protection against lethality than control group (*P* < 0.05). The weight loss and clinical scores reflected the survival data. At the time of the next weight, when only one mouse was left we stopped graphing then. As shown in Fig. 5b, antagomir treatment delayed weight loss compared to control (*P* < 0.05) agomir groups (*P* < 0.05). We also evaluated the clinical scores (weight loss, posture, activity, fur texture, and skin integrity)^33^. The results in Fig. 5c suggested that antagomir-treated tTreg cells protected clinical symptoms (*P* < 0.05 for control and agomir groups both). Interestingly, although agomir treatment promotes cell apoptosis in vitro, agomir treatment did not show significantly difference compared to control group in vivo. We used the same methods in our previous study, which utilized HLA-mismatched tTreg/PBMCs to track tTreg survival in GVHD models^17^. All of the human tTreg cells were undetectable after day 14, but the results in Fig. 5d,e demonstrate that tTreg survival was improved on day 7 and 10.

Thus, knock down of miR-142-3p in tTregs increases in vivo efficacy and can be exploited to improve the efficacy of adoptive Treg therapy for the prevention of human GVHD.

## Discussion

In Treg clinical trials, the researchers are trying to improve cell efficiency from two aspects: (1) developing new methods to improve Treg suppressive function; (2) improving cell expansion and prolonging their survival. Although previous studies suggest that miR-142-3p restricts cAMP production Treg cells by targeting AC9 mRNA, it does provide insights as to how miR-142-3p affects Treg function and whether regulation of miR-142-3p would improve the therapeutic potential of Treg adoptive cell therapy^34,35^. In our study, we developed a new strategy for tTreg efficiency based on miR-142-3p—ATG16L1—FoxP3 axis that revealed: (1) whereas FoxP3 expression remained stable the anti-apoptosis genes (Mcl-1, c-myc) and pro-apoptosis genes (BAX, BID) changed such that tTreg showed more apoptosis status with weaker proliferative ability; (2) miR-142-3p directly targets ATG16L1 mRNA, and in human tTreg cells miR-142-3p negatively regulates an ATG16L1-mediated autophagy effect; (3) knock-down of miR-142-3p upregulates ATG16L1 expression, and these tTreg cells showed higher FoxP3 expression and suppressive function than control tTreg cells; (4) knock-down of miR-142-3p prolonged tTreg survival associated with increased anti-apoptosis and decreased pro-apoptosis gene expression; (5) tTreg cells with attenuated miR-142-3p expression demonstrated better protective effects in a xenogeneic model of GVHD. These findings suggest a new pathway to mediate tTreg function and survival based on miRNA level.

miRNAs are involved in cell development and differentiation^36^. FOXP3 drives miR-142-3p expression in Treg cells and downregulation of miR-142-3p confers heightened Treg suppressor function by increasing the levels of AC9 and cAMP; thus, miR-142-3p might represent a “negative function biomarker” in Tregs^35^. However, freshly isolated tTreg cells show less miR-142-3p expression compared to conventional T cells, which indicates that FOXP3 is not necessary for miR-142-3p expression^37^. In our study, we show that although FOXP3 expression remained high during ex vivo expansion, miR-142-3p increased with time. Interestingly, some studies confirm that miR-142-3p inhibits cell proliferation and induces apoptosis^38–40^, while others demonstrate that miR-142-3p promotes proliferation but is irrelevant in the apoptosis process^41^ and even inhibits apoptosis^42,43^. However these studies did not analyze tTregs. We demonstrate here that miR-142-3p negatively correlates with tTreg cell expansion and survival. In basic and clinical research for tTreg cells, one of the greatest challenges is acquiring enough tTreg cells expanded in vitro for infusion in vivo to prevent or treat immune dysregulation. In the process of these cultures, the proliferative capacity of tTreg cells decline beginning after 10 days from initial stimulation and exhibited more apoptotic status with reduced autophagy in the later days. Though the down-regulation of miR-142-3p, autophagy-mediated anti-apoptosis genes (Mcl-1 and c-myc) and pro-apoptosis genes (BAX and BID) changed, resulting in greater expansion and tTreg cell survival.

Autophagy serves a potential protective role in the PB T cells^44^. Disturbing of autophagy also involves in autoimmune disease and impaired Treg suppressive function^13,45,46^. Thus, we tested ATG16L1 gene, which is a potential target of miR-142-3p and regulates FOXP3 expression in tTreg cells. The results suggest that reflective of the autophagy status, as ATG16L1 mRNA declined over time. However, we cannot exclude the contribution of other targets for miR-142-3p in the control of Tregs such as ATF7IP, CFL2, RAB2, TFG, and CPEB2. In our study, we confirm that miR-142-3p regulates FOXP3 and suppressive function via ATG16L1 pathway. ATG16L1 plays an essential role in autophagy. As a target of miR-142-3p, knock-down of this miRNA results in enhanced ATG16L1, leading to augmented autophagy, better suppressive function in vitro and superior tTreg mediated protection of GVHD lethality after in vivo adoptive tTreg cell transfer.

ATG family are essential in cell survival and apoptosis, modulation of cellular traffic, cell signaling, and autoimmunity^47,48^. Three ATGs have been confirmed to be associated with Treg function: Atg7 or Atg5 deletion leads to loss of Treg cells and Atg16l1 differentially regulates Treg and TH2 cells to control intestinal inflammation^22,49^. ATG7 has been shown to contribute to Treg cell survival and lineage stability though Mtorc1 pathway^49^. Based on our findings, ATG16L1 also regulates autophagy-related pro/anti apoptosis genes and up-regulation of ATG16L1 is beneficial for Treg survival.

Treg adoptive cellular therapy has been shown to ameliorate autoimmune disease, graft rejection and GVHD. Translating adoptive tTreg therapy to humans has proceeded slowly due to challenges in maintaining high expression of Foxp3 in cultured cells, overall yield, and in vivo persistence of tTregs in GVHD patients^50,51^. In this study, we have shown that nanoparticle-mediated delivery of miR-142-3p antagomir to in vitro expanded tTreg is an easy and efficient way of overcoming these challenges, which may be useful in clinical applications.

In summary, we demonstrate that miR-142-3p—ATG16L1—FOXP3 pathway plays a vital role in tTreg expansion, survival and function in vivo and vitro, and focusing on miRNA might become a new starting point in Treg clinical trials.
